# Implementation of a Novel Structured Social and Wellness Committee in a Surgical Residency Program: A Case Study

**DOI:** 10.3389/fsurg.2017.00014

**Published:** 2017-03-13

**Authors:** Kathryn E. Van Orden, Stephanie D. Talutis, Joanna H. Ng-Glazier, Aaron P. Richman, Elliot C. Pennington, Megan G. Janeway, Douglas F. Kauffman, Tracey A. Dechert

**Affiliations:** ^1^Department of Surgery, Boston Medical Center, Boston University School of Medicine, Boston, MA, USA; ^2^Division of Pediatric Surgery, Ann & Robert H. Lurie Children’s Hospital of Chicago, Chicago, IL, USA; ^3^Northwestern University Feinberg School of Medicine, Chicago, IL, USA

**Keywords:** self determination, social wellness, surgical residency, basic needs, belongingness, competence, autonomy

## Abstract

This article provides a theoretical and practical rational for the implementation of an innovative and comprehensive social wellness program in a surgical residency program at a large safety net hospital on the East Coast of the United States. Using basic needs theory, we describe why it is particularly important for surgical residency programs to consider the residents sense of competence, autonomy, and belonging during residence. We describe how we have developed a comprehensive program to address our residents’ (and residents’ families) psychological needs for competence, autonomy, and belongingness.

## Background

Humans have an innate, psychological necessity to build and sustain supportive relationships with one another. This psychological need to affiliate is abundantly described throughout the motivational sciences literature (1–5). Maslow (3) argued that along with basic physiological (e.g., food and water) and safety (e.g., shelter) needs, developing a sense of belongingness with others is a fundamental human necessity that must be satisfied before psychological and intellectual growth can occur. Likewise, Ryan and Deci (4) maintain that those who feel connected to others will be more psychologically healthy than those who feel disconnected. From this perspective, when individuals are able to satisfy their desire for affiliation they feel psychologically balanced which results in further motivation to concentrate on intellectual development.

The demands of surgical residency can put a strain on the young physician’s well-being. Based on the research described above (1–5), one can argue that residents who build and sustain supportive relationships among co-residents and faculty will be more psychologically healthy and will be more likely to value their training. If this holds true, medical educators hold uniquely powerful positions in that their ability to create long-term relationships with residents may be a foundation for developing and sustaining resident motivation and intellectual drive. Thus, the capacity to facilitate supportive and meaningful relationships should be a critical prerequisite for establishing an effective surgical residency. This article describes the Boston Medical Center general surgery residency program’s efforts to foster such a program.

## Introduction

Surgical residency is a challenging time. Residents are required to dedicate nearly all of their resources (cognitive, behavioral, emotional, and physical) to developing clinical competencies, often at the expense of maintaining their own physical and psychological health. At Boston Medical Center, we believe strongly that helping residents and their families feel psychologically affiliated with their peers and mentors is the first step toward meeting our goal of more emotionally healthy residents who are able to put forth more energy to become superior surgeons. This belief is also consistent with ACGME and ABS’s General Surgery Milestones Project which lists *maintenance of physical and emotional health* as one of just seven *Practice Domains* that residents are assessed on during their training. Thus, over the past several years, we have developed a comprehensive program designed to help both our residents and their families feel connected to one another.

The general surgery Social and Wellness Committee was established at Boston Medical Center in 2011. Our principal objectives are to improve departmental communication and morale and to enhance the surgical residency recruitment process. In addition, we serve as a more formal support system for residents and faculty during major life events. We organize monthly social gatherings and larger quarterly events. Further, the Social and Wellness Committee organizes several resident-led activities for the recruitment process. Our multidimensional efforts continue to build strong relationships among residents and faculty, sustain resident motivation, and improve resident recruitment. This, in turn, has strengthened our residents’ interpersonal and communication skills, one of the six core competencies, with one another, surgical advanced practitioners, fellows, faculty, staff, and consultants.

## Our Programs

In September 2011, with low resident morale and a strong desire by a group of residents and faculty to change the attitude of the Boston Medical Center (BMC) general surgery residency program, the Social and Wellness committee was formed. The BMC general surgery residency is comprised of five general surgery residents per year training in a large 492 bed academic, safety-net hospital. The committee is comprised of one resident representative from every training level, one faculty advisor, and one doctoral education advisor. Over the last 5 years, the committee has developed several programs within our surgical residency aimed at satisfying our principle objectives.

Surgical residency can be socially isolating. Through the match system, residents oftentimes move to a new and unfamiliar place far from family. They begin working rigorous hours and no longer have the luxury of vacations during each holiday season. Time for family and friends becomes limited and residents can feel alone. In an attempt to thwart this loneliness and to foster connectedness, we have organized monthly social gatherings at local venues. We have also organized larger, quarterly events, such as kickball games, cookouts, bowling, and holiday dinners. In addition to our monthly and quarterly social events, we have started an orientation initiative for new interns as well as each rising PGY year. This was created in an effort to foster collaborative working relationships and to aid in transition from year to year. We have organized gatherings among an outgoing PGY class and their incoming counterparts to answer questions and provide insight and guidance into the expectations for the upcoming year. In addition, during orientation week in June, the interns, residents, faculty, and staff take a guided Duck Boat tour of Boston to build relationships with the new interns. Each of these events creates opportunities for residents and faculty to spend time together and build relationships in order to foster a feeling of connectedness among the group.

Surgical residency can place a significant strain on families, particularly families with young children. *Surgery Kids* is a program started by the Social and Wellness Committee designed to connect those within the department as well as within the surgical subspecialties who have families with young children. *Surgery Kids* organizes quarterly activities for residents and their families throughout the community. Events in the past have included a visit to the zoo, a trip to a local children’s museum, and a picnic in the park. We are confident that these activities help our residents and their families feel connected to the surgery department at BMC and more importantly to each other.

Surgical residency can pose substantial challenges for women. Despite significant advances in gender equality within the field of surgery, women are often still striving for acceptance and equal opportunities within a historically male-dominated profession. In addition, studies suggest that women are up to twice as likely as men to leave surgical training. *WOW*, named after the women ordinance workers of World War II, is a woman in surgery group started by the Social and Wellness Committee to provide an outlet for women within the general surgery department as well as the surgical subspecialties of orthopedic surgery, plastic surgery, urology, neurosurgery, and otolaryngology. The group gets together quarterly for social, professional, and emotional support. Events in the past include a wine and cheese night as well as a trip to a spa. *WOW* provides a safe place for women to discuss and share their experiences as well as to build and strengthen their relationships.

Surgical residency is not an isolated event. Life, both good and bad, continues even as residents spend much of their time at work in the hospital. The Social and Wellness Committee has expanded in recent years to become a formal support system during these significant life events. From the birth of a child to the death of a family member, the Social and Wellness Committee provides congratulations or support. We email birth announcements; we send get well cards and care packages to those with significant illness; and we send flowers to those who have lost a loved one. Each year we are able to collect donations from residents and faculty to be able to show this dedication to our colleagues. These small gestures remind the residents that they are not alone and that they are supported.

Recruitment for surgical residency is a complex process. Through the aid of the Social and Wellness Committee, residents have become significantly more involved in the recruitment process through the pre-interview dinner, interview day tours, and post-interview candidate evaluations. The Committee hosts the pre-interview socials and is proactive in ensuring high resident attendance. These events, in turn, are rated very highly by applicants in post-interview surveys, with many candidates commenting on the strong resident presence. Robust resident involvement reflects to our applicants both the sense of connectedness our residents feel to one another as well as the investment we have in recruitment of not only competitive but also well rounded applicants who are compatible with our program.

## Conclusion

At Boston Medical Center, the general surgery residency strives to train excellent surgeons. Certainly, ensuring that our residents have every opportunity to meet milestones, exceed clinical competencies, pass boards, and begin successful careers is fundamental to our training program. Just as importantly, however, we believe that maintaining one’s well-being and work-life balance is crucial. We feel strongly that by committing our surgical education program to satisfying our residents’ needs for affiliation in a planned and productive manner, we are able to train emotionally healthy surgical residents. Moreover, we believe that creating habits of wellness in training will provide the foundation necessary to maintain physical and emotional health throughout one’s career, preventing physician burn out and fostering fulfilling and productive practices. This desire for wellness is shared by our institution which has created its own Wellness Program for employees. In the future, we hope to satisfy Boston Medical Center’s growing interest in mental health by aiding other residency programs in developing a Social and Wellness Committee and by securing dedicated funding for resident wellness initiatives.

Humans have an inherent need to belong. This sense of relatedness to one another must be fulfilled prior to further psychological and intellectual development. Surgical residency is an exquisitely challenging and often socially isolating time in one’s life. Currently, residency programs provide few resources aimed at improving wellness and increasing connectedness among trainees. Over the last 5 years, we have developed the Social and Wellness Committee to provide programs and initiatives within our surgical residency that have had a tremendously positive influence on our surgical residents, fellows, advanced practitioners, and faculty. At Boston Medical Center, our Social and Wellness Committee has improved communication, connectedness, and morale; has sustained resident motivation; and has bettered resident recruitment. In summary, this committee has begun to show us that connecting to one another is far more important than simply building friendships; it has a lasting, motivational effect on residents as well as faculty.

## Author Contributions

All authors contributed equally to this project. DK, KO, ST, and MJ were primarily responsible for the write-up. EP, JN-G, AR, and TD were responsible for the development and implementation of the educational programming described in the article.

## Conflict of Interest Statement

The authors declare that the research was conducted in the absence of any commercial or financial relationships that could be construed as a potential conflict of interest.
